# Geography and social distribution of malaria in Indonesian Papua: a cross-sectional study

**DOI:** 10.1186/s12942-016-0043-y

**Published:** 2016-04-12

**Authors:** Wulung Hanandita, Gindo Tampubolon

**Affiliations:** Cathie Marsh Institute for Social Research (CMIST), University Manchester, Oxford Road, Manchester, M13 9PL UK

**Keywords:** Malaria, Map, Papua, Indonesia, Bayesian, Spatial, Multilevel

## Abstract

**Background:**

Despite being one of the world’s most affected regions, only little is known about the social and spatial distributions of malaria in Indonesian Papua. Existing studies tend to be descriptive in nature; their inferences are prone to confounding and selection biases. At the same time, there remains limited malaria-cartographic activity in the region. Analysing a subset (N = 22,643) of the National Basic Health Research 2007 dataset (N = 987,205), this paper aims to quantify the district-specific risk of malaria in Papua and to understand how socio-demographic/economic factors measured at individual and district levels are associated with individual’s probability of contracting the disease.

**Methods:**

We adopt a Bayesian hierarchical logistic regression model that accommodates not only the nesting of individuals within the island’s 27 administrative units but also the spatial autocorrelation among these locations. Both individual and contextual characteristics are included as predictors in the model; a normal conditional autoregressive prior and an exchangeable one are assigned to the random effects. Robustness is then assessed through sensitivity analyses using alternative hyperpriors.

**Results:**

We find that rural Papuans as well as those who live in poor, densely forested, lowland districts are at a higher risk of infection than their counterparts. We also find age and gender differentials in malaria prevalence, if only to a small degree. Nine districts are estimated to have higher-than-expected malaria risks; the extent of spatial variation on the island remains notable even after accounting for socio-demographic/economic risk factors.

**Conclusions:**

Although we show that malaria is geography-dependent in Indonesian Papua, it is also a disease of poverty. This means that malaria eradication requires not only biological (proximal) interventions but also social (distal) ones.

## Background

**Fig. 1:**
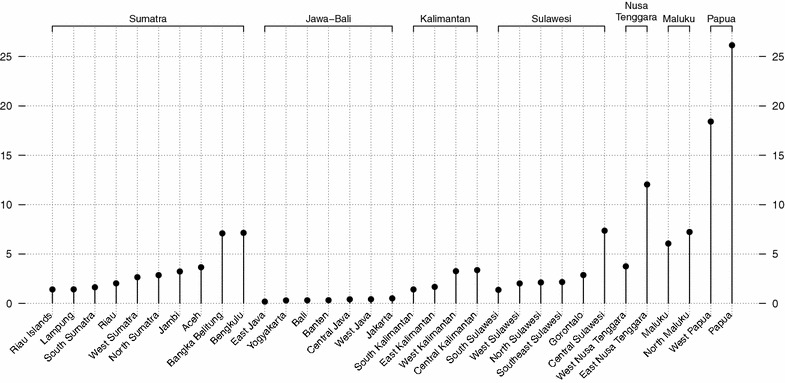
Malaria prevalence in 33 Indonesian provinces in 2007 (%), sorted by island group’s longitude (*left to right* = west to east, low to high prevalence; *source* [13])

**Fig. 2:**
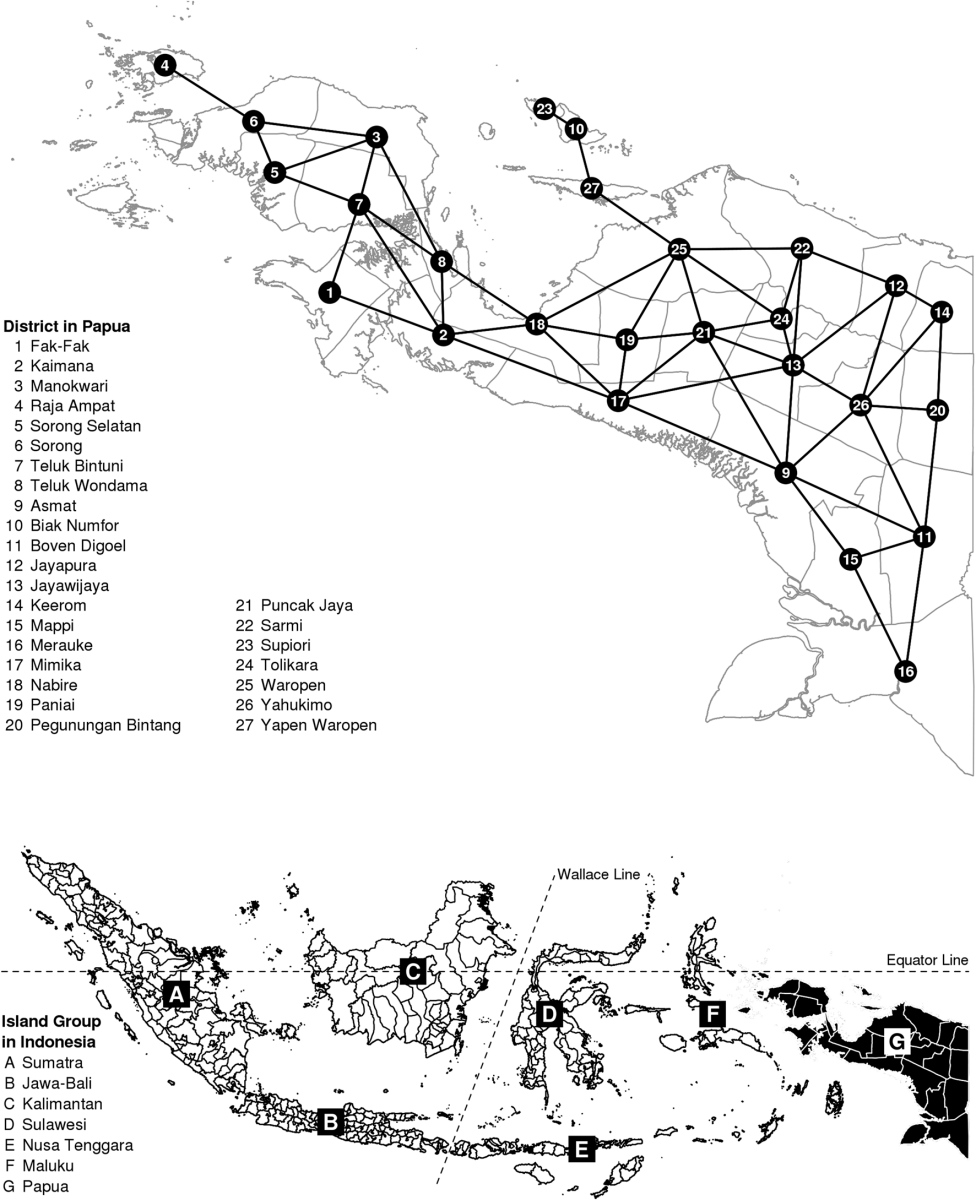
Setting of the study

Malaria, a mosquito-borne infectious disease that inflicts devastating health [1, 2] and economic [3–5] costs on society, remains a major problem in Indonesian Papua [6] (Fig. 1). This region of mixed-parasite endemicity is located in the easternmost part of the Indonesian archipelago (Fig. 2) and is classified by the World Health Organization (WHO) as hyper-endemic area with annual parasite incidence (API) greater than 10 % (nationwide ${\mathrm {API<1\,\%}}$; [7]) and parasite prevalence (PP) as high as 50–75 % (nationwide ${\mathrm {PP<1\,\%}}$; [8]). Malaria accounts for a considerable proportion (15–34 %) of total hospital workload in the region [9]; mortality due to severe anaemia [10] as well as multi-drug resistance with high rate of therapeutic failure (65–95 %) have been documented [11, 12]. In 2007, the Ministry of Health of the Republic of Indonesia [13] estimated that the infectious disease was prevalent among one-fifth (22.25 %) of the Papuan population—a figure that is seven times higher than the national average (Fig. 1). Perhaps nothing can highlight the seriousness of this situation better than the fact that while malaria prevalence for the whole Indonesian archipelago decreased from 2.9 % in 2007 [13] to 1.9 % in 2013 [14], the figure for Papua actually increased to 24 % over the same period.

Defeating malaria is certainly a high priority for Indonesian policy makers; they have not only set the year 2030 as the deadline for malaria elimination in the country [15] but have also entrusted local Papuan administrators with the responsibility for preventing and combating endemic diseases through the enactment of the 2001 Papua Special Autonomy Law No. 21 [16]. Notwithstanding these political commitments, challenges to disease control in Papua remain. Principal among them is that the spatial distribution of malaria, which is vital for guiding efficient and equitable allocation of the limited resources available for intervention, is still understudied. To date, the only risk map available for the region is the one produced by the Malaria Atlas Project (MAP), which, while informative, was unfortunately based on community blood surveys carried out in non-randomly selected locations [17, 18]. Moreover, because the risk estimate in the existing malaria maps is presented as a continuous surface obtained from geostatistical models that are blind to political boundaries, there is no straightforward way to obtain a single summary [19] for each local administrative unit in Papua. Policy makers in now-decentralised Indonesia [20] are therefore deprived of an intuitive tool for prioritising development projects or other forms of intervention that are funded by transfers from central to local governments (the *Kabupaten*/*Kota* or the district/municipality).

This scarcity of malaria-cartographic activity is further complicated by the fact that, unlike in Africa, the social and environmental determinants of malaria in Papua have not yet been thoroughly examined. Existing knowledge—that the risk of contracting the disease seems to be higher among non-native Papuans [12, 21], children and young adults [10], as well as rural [21] and lowland dwellers [10]—was in fact elicited from simple descriptive or bivariate analyses performed on small community or facility samples that are prone to both confounding and selection biases. So, although Papua is reputed to be one of the most malaria-ridden regions in the world [22], to date, only little is known about the social and spatial aspects of the disease. Without precise knowledge of where in Papua malaria strikes and which population subgroup it hits the hardest, it is likely to be difficult for Indonesian policy makers to meet the 2030 elimination target on time.

**Fig. 3:**
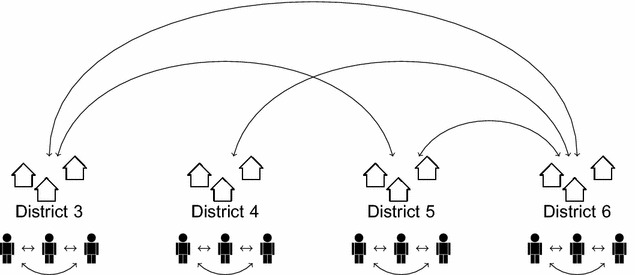
Illustration of hierarchical and spatial dependence

Analysing large population data (N = 22,643) from the National Basic Health Research 2007 (*Riset Kesehatan Dasar*; [13]), this study aims to address these gaps. Through the application of a Bayesian hierarchical modelling technique that accounts for both the nesting of individuals within districts (vertical dependence) and the spatial autocorrelation among these areas (horizontal dependence; see Fig. 3), this paper seeks (1) to quantify the district-specific risk of malaria in Papua and (2) to understand how socio-demographic/economic factors measured at individual and district levels are associated with an individual’s probability of contracting the disease. The novelty of this paper is threefold. First, in using randomly sampled population data from Indonesia’s largest public health study, this paper avoids the problem of confounding and selection biases that beset earlier studies mentioned above. Second, through its spatial analysis of irregular lattice data, this study is able to deliver a single risk summary for each district and municipality in Papua, which is the lowest autonomous administrative unit in the Indonesian political system. Finally, the present study is also distinguished from others in its multilevel analysis of individual and contextual determinants of malaria, avoiding ecological fallacy [23, 24].

 The remainder of this paper is structured as follows. The next section describes the study site, data, measures and modelling techniques. Third section presents the results. Fourth section discusses the findings. Finally, fifth section concludes.

## Methods

### Study site

This study was carried out in the western half, or the Indonesian side, of the New Guinea island, commonly referred to as the *Papua* or *Irian Jaya* region among Indonesians (Fig. 2). Lying between latitudes 0–9$^{\circ }$ South and longitudes 124–141$^{\circ }$ East, the climate of Papua is entirely tropical, with a dry season typically occurring from April–October and a wet season from October–April. Most of Papua’s land area is covered by forests. Apart from a mountain range stretching more than 1500 km from the west to central east of the island, the topography of Papua is shaped by the extensive presence of swamps, wetlands, mangroves, savannah grasslands, lakes and rivers. Rain persists throughout the year (150–270 days of rain per year), yielding 2000–3000 mm of annual rainfall [25, 26]. The average humidity is 80–90 % while the average temperature is about $26^{\circ }$ Celsius, with an average maximum of $30^{\circ }$ and an average minimum of $22^{\circ }$ [25, 26].

**Fig. 4:**
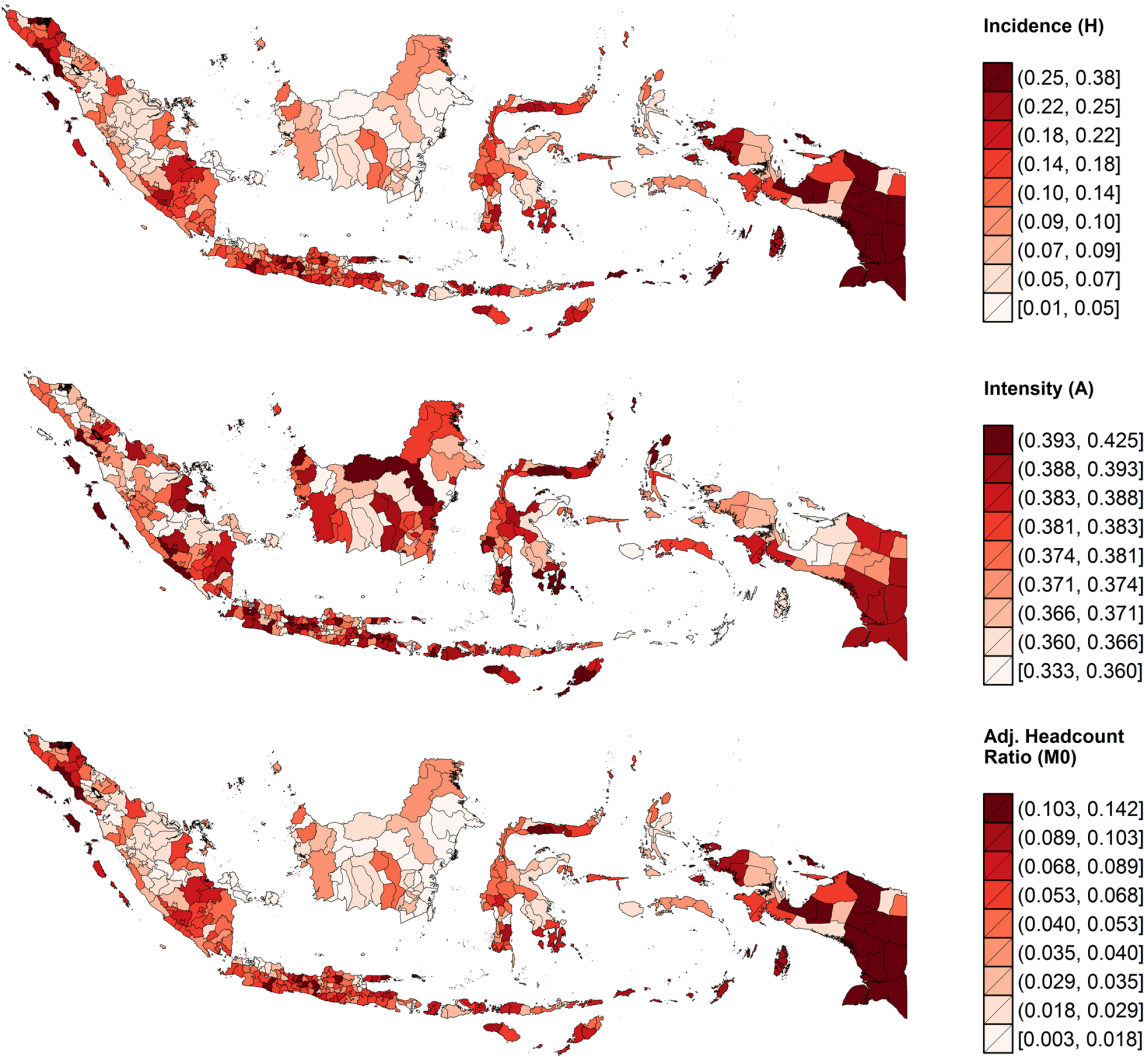
An Indonesian version of the Alkire-Foster multidimensional poverty index [88] for the year 2013 (*source* [28])

According to the latest census conducted in 2010 [27], the population of Papua is 3.6 million (2 % of Indonesia’s population) living in an area of 420,540 ${\text {km}}^{2}$ (22 % of the country’s land area), with a population density of just 9 persons per square kilometre (the lowest in Indonesia). As many as 70–75 % of Papuans live in rural areas [27]. Despite hosting one of the planet’s largest gold mining operations (the Grassberg mine in Mimika district), Papuan society is plagued by poverty and under-development. As shown in Fig. 4, Hanandita and Tampubolon [28] estimate that approximately a quarter of Papuan adults aged 18 and older were multidimensionally poor in 2013; collectively, they were subjected to about 10 % of the total deprivation (in terms of income, illness episodes, morbidity, schooling and literacy) potentially experienced by all adult Indonesian that year. The combination of geographic features, climate conditions and extreme poverty provides a suitable environment for malaria transmission, both biologically and socially [4, 29, 30].

### Data

We analyse data drawn from the National Basic Health Research (*Riset Kesehatan Dasar*, Riskesdas) 2007. Involving 987,205 individuals from 258,366 households in 440 districts, Riskesdas is the largest public health study ever conducted by the Ministry of Health of the Republic of Indonesia [13]. For our analysis, we sampled individuals of all ages living in Papua, yielding a total sample size of 22,643 individuals.

Information on each respondent’s malaria status, age, sex, use of insecticide-treated net (ITN), and urban/rural residential location is available from the Riskesdas 2007 dataset. However, because the household consumption expenditure module was not administered to survey respondents living in a number of Papuan districts, we are unable to include a measure of individual income. Instead, we obtain a measure of wealth in the form of each district’s median per capita household consumption expenditure [31, 32], computed from the National Socio-economic Survey (*Survei Social Ekonomi Nasional*, Susenas) 2008 dataset. We also obtain additional information on districts’ median household elevation (as a proxy for temperature and precipitation; [33]) and the proportion of districts’ populations living in or near forest (as a proxy for forest density). This contextual information is derived from the Village Census (*Potensi Desa*, Podes) 2008 dataset that covers all 75,410 villages across the Indonesian archipelago.

Spatial polygons and the associated political boundary data are obtained from the freely-accessible GADM database of global administrative area (www.gadm.org; [34]). Originally, there were 29 districts and municipalities in Papua in 2007, but due to the lack of spatial polygons for *Kota Sorong* and *Kota Jayapura* municipalities, we have no choice but to regroup study participants living in these locations with those living in *Kabupaten Sorong* and *Kabupaten Jayapura* districts, respectively. This will not come as a surprise to researchers analysing data from Indonesia. Parmanto et al. [35] write at some length about both the poor quality of the country’s spatial data and the government’s slow process of updating administrative boundaries.

Of the 22,643 individuals selected as our study sample, 871 (3.8 %) have missing values and are thus excluded from the subsequent multivariate modelling exercise. This data-cleaning procedure produces a final complete-case sample size of 21,772 individuals, corresponding to 96.2 % of the original Papuan sample of the Riskesdas 2007 study. Informed consent was obtained prior to data collection; study participants’ confidentiality was strictly protected by means of anonymisation [13].

### Measures and a priori expectations

The outcome variable, namely the individual’s malaria status, is coded as a binary variable whose value equals one (malaria-positive) if, within the past month, the study participant had been diagnosed with laboratory-confirmed malaria*,* suffered from high fever accompanied by chills, sweating, or headache, or took anti-malarial drugs [13]. Age is treated as a 7-category ordinal variable indicating the respondent’s age group (0–4, 5–14, 15–24, 25–34, 35–44, 45–54, and 55+). Sex, ITN use, and urban/rural residential location are each entered as a dummy variable representing female individuals, respondents who slept under an ITN the night prior to data collection, and those living in rural areas, respectively.

The three contextual variables are operationalised in the following way. Because no district has a median elevation between 200 and 1200 m, median household elevation is treated as a dummy variable indicating whether the majority of the district’s population lives in lowland ($\le$200 m above sea level) or highland ($\ge$1200 m). The proportion of a district’s population living in or near forest is multiplied by a factor of 10 and used as a continuous variable. For ease of interpretation as well as for capturing a possible non-linear relationship, district median income is split into quintiles before being entered into the statistical model described next as a set of four dummy variables indicating the relative wealth of each district in Papua. With this set up, we then set the reference individuals (the intercept) in the model to represent urban, ITN non-user, male infants living in the poorest, least-densely-forested, highland district.

A priori, we expect that the chance of contracting malaria will be relatively high among individuals living in rural areas and in poor, densely forested, lowland districts of Papua. This is because the extant literature has already hinted that:

- there is an inverse relationship between temperature (hence altitude and latitude) and the length of the *plasmodium* growth-cycle [4, 30, 36, 37];
- the micro-climate of forests enhances *anophelines* breeding sites and prolongs their survival as adults [38, 39];
- the pollution and high population density of urban areas entail poor mosquito habitats and low biting frequency [29, 40]; and that
- poverty creates conditions (poor housing, lack of health knowledge, negative health behaviours) that favour the spread of infectious diseases and restrict access to prevention and treatment [41–43].

We also expect that the probability of being malaria-positive will be high among those who do not sleep under ITN due to the lack of a physical barrier separating them from the mosquitoes [44]. Mendis et al. [45] suggest that the ‘male rather than female’ as well as the ‘working age rather than infant or elderly’ infection patterns that are commonly found in South East Asian countries are unlikely to hold in high endemicity areas such as Papua. Studies from Peru [46], Bangladesh [41, 47], Malawi [48], Gambia [49], and India [50] present conflicting evidence regarding the age and gender distributions of malaria.

### Modelling techniques

To predict the malaria status of individual *i* living in district *j*, a Bayesian generalised linear model (GLM) with random effects is fitted [51, 52]. We assume, for the data model, that a person’s malaria status arises from the realisation of a Bernoulli trial with the probability of success (malaria-positive) $\pi _{ij}$ as shown in Eq. 1. In the process model (Eqs. 2 and 3), we take the logit of $\pi _{ij}$ and model it as a linear combination of observed individual $(x_{ij})$ and contextual $(x_{j})$ characteristics with parameter vector $\beta$ plus an unobserved district-specific effect $\xi _{j}$. The $\xi _{j}$ can be intuitively understood as random intercepts indicating how much the risk of contracting malaria in each district varies from the island’s average $(\beta _{0})$ after accounting for the effects of all observed covariates $(\sum _{p=1}^{P}\beta _{p}x_{pij})$. This district-specific effect is further decomposed additively into its spatially structured $(u_{j})$ and unstructured $(v_{j})$ components, which, in combination, are capable of incorporating the dependency structure of spatially correlated multilevel data (Fig.  3) into the modelling process [53].

1 $$\begin{aligned} y_{ij}\sim {\mathrm {Bernoulli}}(\pi _{ij}) \end{aligned}$$

2 $$\begin{aligned} {\text {logit}}(\pi _{ij})= (X\beta )_{ij}+\xi _{j} \end{aligned}$$

3 $$\begin{aligned} \log \left[ \frac{\pi _{ij}}{1-\pi _{ij}}\right]= \beta _{0}+\sum _{p=1}^{P}\beta _{p}x_{pij}+u_{j}+v_{j} \end{aligned}$$

4 $$\begin{aligned} \beta\sim {\mathrm {Normal}}(0,10^{-4}) \end{aligned}$$

5 $$\begin{aligned} u_{j}|u_{k},j\ne k,\tau _{u}\sim {\mathrm {Normal}}\left( \frac{1}{\mathcal {N}_{j}}\sum _{j\sim k}u_{k},\frac{1}{\mathcal {N}_{j}\tau _{u}}\right) \end{aligned}$$

6 $$\begin{aligned} v_{j}\sim {\mathrm {Normal}}(0,\tau _{v}) \end{aligned}$$

7 $$\begin{aligned} \tau _{u}\sim {\mathrm {Gamma}}(10^{-3},10^{-3}) \end{aligned}$$

8 $$\begin{aligned} \tau _{v}\sim {\mathrm {Gamma}}(10^{-3},10^{-3}) \end{aligned}$$

The body of epidemiology and parasitology research [54–57] suggests that either ignoring *spatial heterogeneity* (vertical dependency) induced by the clustering of individuals within areas of residence or omitting *spatial autocorrelation* (horizontal dependency) among adjacent areas could result in severely underestimated uncertainty with respect to the estimation of regression parameters; in some cases, it could even result in biased estimates (see [24, 58, 59] for elaboration in general context). Chirombo et al. [48] suggest that, technicalities aside, the spatially structured random effect $u_{j}$ plays a crucial role in capturing the unmeasured between-area variation in access to health facilities and interventions, while the unstructured component $v_{j}$ is useful for absorbing the unobserved level of immunity to malaria that varies randomly across the locations. In general, one may view this random effects specification as a method of incorporating the effects of unmeasurable natural and social features that transcend political borders.

Prior distributions for the unknown random parameters are specified as follows. The regression parameter $\beta$, which determines how the risk of malaria is distributed across socio-demographic/economic strata in Papua, is assigned a diffuse normal prior with mean zero and extremely low precision (Eq. 4). The spatially structured random effect $u_{j}$ is given a conditional autoregressive (CAR) prior [60] whose mean and precision depend on the structure as well as the number $({\mathcal {N}}_{j})$ of the adjacent first-order neighbours $(j\sim k)$ of each district (Eq. 5). The binary adjacency matrix for this prior is constructed using queen contiguity criteria [61]; the implied neighbourhood graph is shown in the top panel of Fig.  2. This Markov random field (MRF) approach to spatial modelling has been recently applied to analyses of malaria in Malawi [48, 62], antenatal care in Kenya [63], and childhood health outcomes in Tanzania, Malawi and Zambia [64, 65], among others. Best et al. [66] and Kauermann et al. [67] report the relatively good performance of the MRF model in comparison to other spatial-statistical and spatial-econometrics models. For the spatially unstructured random effect $v_{j}$, a typical normal prior with an exchangeable structure is assumed (Eq. 6). We then choose ${\mathrm {Gamma}}(0.001,0.001)$, a proper approximation of a Jeffreys non-informative prior [68], as the default prior for the precisions of $u_{j}$ and $v_{j}$ (Eqs. 7 and 8) although later (in Figs. 6, 8), we also conduct sensitivity analysis using alternative ${\mathrm {Gamma}}(a,b)$ hyperpriors [69].

Marginal posterior distributions of model parameters are obtained using integrated nested Laplace approximation (INLA), which is not only a valid but also an efficient alternative to the commonly used Markov Chain Monte Carlo (MCMC) simulation method [70–73]. To facilitate interpretation, we derive quantities that are of particular interest to policy makers, such as the odds ratio $(\exp [\beta ],\;\exp [\xi _{j}])$, the probability of excess risk $(\Pr [\exp \{\xi _{j}\}>1|{\mathbf {y}}]=\Pr [\xi _{j}>0|{\mathbf {y}}])$, the baseline probability of malaria infection $({\text {logit}}^{-1}[\beta _{0}+\xi _{j}]={\text {logit}}^{-1}[\beta _{0j}])$, as well as the fraction of district-level variance attributed to spatial autocorrelation $(\phi =\sigma _{u}^{2}/[\sigma _{u}^{2}+\sigma _{v}^{2}])$. A deviance information criterion (DIC; [74]) is used to evaluate the performance of the full model against the null. Where a density curve is not shown, we summarise the posterior distribution of a model parameter using its mean, accompanied by the 95 % credible interval.

## Results

### Descriptive and bivariate analysis

The second column in Table  1 shows the univariate description of the sample. Confirming the official tabulation released by the Ministry of Health [13], about one-fifth of study participants (21.06 %) reported they had been infected with malaria. In the sample, sex appears to be distributed equally; about 60 % of study participants are of working age ($\ge$15 years old); the vast majority (78 %) of them are ITN non-users or rural dwellers. With respect to elevation, it appears that only 6 out of 27 districts (22.22 %) are categorised as highland districts ($\ge$1200 m). It turns out that about half ($\hat{p}=0.52$; SD = 0.24) of Papuan population live in the vicinity of forest; and assuming a historical 1 US Dollar (USD) to 10,000 Indonesian Rupiah (IDR) exchange rate, the district median per capita daily consumption expenditure is around USD 1.30 (SD = 0.50).

**Table 1:** Descriptive and bivariate analysis

Variable	Summary statistic	Unadjusted odds ratio [95 % CI]
*Individual characteristics (N = 22,643)*		
Malaria status		
No	78.94 %	
Yes	21.06 %	
Sex		
Male	49.62 %	1.00
Female	50.38 %	0.96 [0.90, 1.02]
Age group		
0–4 (Infant)	12.39 %	1.00
5–14	26.84 %	0.93 [0.83, 1.04]
15–24	14.36 %	0.90 [0.80, 1.02]
25–34	16.41 %	1.02 [0.90, 1.15]
35–44	14.98 %	1.01 [0.90, 1.15]
45–54	9.40 %	1.03 [0.90, 1.18]
55+	5.62 %	1.15 [0.98, 1.35]
Sleep under ITN		
No	78.62 %	1.00
Yes	21.38 %	1.15 [1.07, 1.25]
Residential location		
Urban	22.14 %	1.00
Rural	77.86 %	1.43 [1.31, 1.55]
*District characteristics (N = 27)*		
Median household elevation		
Highland ($\ge$1200 m)	22.22 %	1.00
Lowland ($\le$200 m)	77.78 %	1.65 [1.51, 1.79]
Proportion living in or near forest	0.52 ± 0.24	1.07 [1.05, 1.08]
Median income		
Quintile 1 (poorest)	22.22 %	1.00
Quintile 2	18.52 %	1.41 [1.27, 1.57]
Quintile 3	22.22 %	0.95 [0.87, 1.04]
Quintile 4	18.52 %	0.91 [0.82, 1.01]
Quintile 5 (richest)	18.52 %	0.72 [0.66, 0.80]

The magnitude of bivariate associations between an individual’s malaria status and its predictors is presented in the rightmost column of Table 1. Confirming conventional wisdom, the analysis suggests that Papuans living in rural area or in poor, densely forested, lowland districts are at a relatively higher risk of contracting malaria than their counterparts in urban or highland settings. Age and gender do not seem to explain much of the between-individual variability in disease prevalence, although there appears to be a weak indication for the presence of a threshold effect in the relationship between age and malaria status. Contradicting a priori expectation, the analysis shows that the odds of being malaria-positive increase with respondents’ use of ITN on the night prior to data collection. Of course, it is prudent to note that this paradoxical finding could arise from our application of a simple GLM that neither adjusts for confounding nor accounts for the complex dependency structure of the data. Whether this unexpected ITN effect is simply a statistical artefact is to be tested in the multivariate analysis presented next.

### Multivariate analysis

**Fig. 5:**
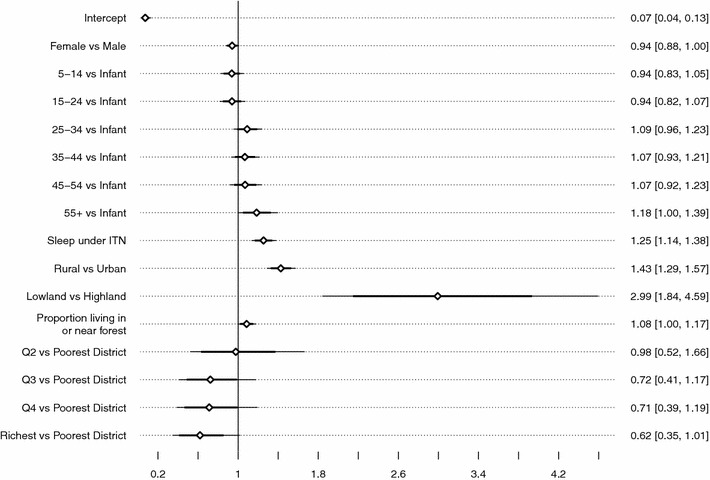
Posterior means of adjusted odds ratio and their 80 and 95 % credible intervals

Figure 5 displays the confounding-adjusted odds ratios (diamond) along with their 80 % (bold line) and 95 % (fine line) credible intervals. The most striking feature of the analysis is that the odds of contracting malaria for individuals living in lowland districts versus those in highland districts have doubled from 1.65 (95 % CI 1.51–1.79) in the simple bivariate model to 2.99 (95 % CI 1.84–4.59) in the multivariate model. Living in a rural area (OR 1.43, 95 % CI 1.29–1.57) and in a densely forested district (OR 1.08, 95 % CI 1.00–1.17) are both associated with higher odds of being malaria-positive; their posterior means (credible intervals) do not, however, vary much from those obtained from the previous bivariate model. The analysis also makes the socio-economic gradient of malaria prevalence in Papua much clearer. The odds of being infected with malaria seem to follow a non-linear, monotonically decreasing function of district median income such that individuals living in the richest 20 % of districts have 38 % lower odds of being malaria-positive, holding all other factors constant. The multivariate analysis also presents evidence of the existence of a threshold effect in the relationship between age and malaria status, because only the elderly (55+ age group) seem to have a distinctively elevated risk of malaria. In addition, the sex difference is now more precisely estimated, with female individuals having 4 % lower odds than their male counterparts. After controlling for all of these, however, we still find an unexpected positive ITN effect, with study participants who slept under a bed-net estimated to have 25 % higher odds of contracting malaria. We discuss plausible explanations for this in the discussion section.

**Fig. 6:**
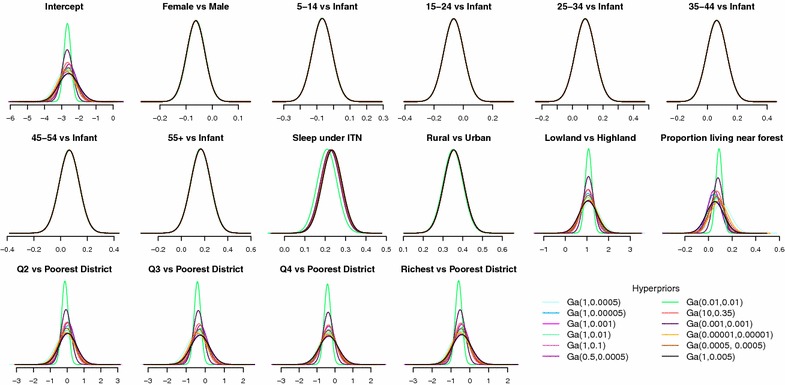
Posterior density of fixed effects coefficients $(\beta )$ under some alternative hyperprior specifications, logit scale

Table 2 compares the performance of the fully specified model against the null model. Clearly, the full fits better than the null, as its improvement in terms of model deviance $(\bar{D})$ far outweighs the increased model complexity (*pD*), leading to a 94.36 point smaller DIC statistic. The covariates seem to have a strong explanatory power; their inclusion into the model leads to a 71 % reduction in the between-district variability of malaria prevalence $(\sigma _{u}^{2}+\sigma _{v}^{2})$. These covariates account for a disproportionately larger proportion of the spatially unstructured between-district variability $(\sigma _{v}^{2})$ than the structured one $(\sigma _{u}^{2})$, which, in turn, inflate the proportion of variance attributed to spatial autocorrelation $(\phi )$ from just 4 % in the empty model to 32 % in the full model. In Fig. 6, we test the sensitivity of regression parameters with respect to the specification of alternative Gamma hyperpriors. Results show that the posteriors are robust to the choice of commonly suggested hyperpriors, albeit with some degree of variation around the width of the credible intervals of the intercept and contextual determinants. Nevertheless, since their means, medians and modes are all very close, the interpretation above remains

**Table 2:** Summary of model fit

Statistic	Null model	Full model
$\bar{D}$	20,656.42	20,553.76
*pD*	26.59	34.89
DIC	20,683.01	20,588.65
$\sigma _{u}^{2}+\sigma _{v}^{2}$	0.76	0.22
$\phi$	0.04	0.32

.

**Fig. 7:**
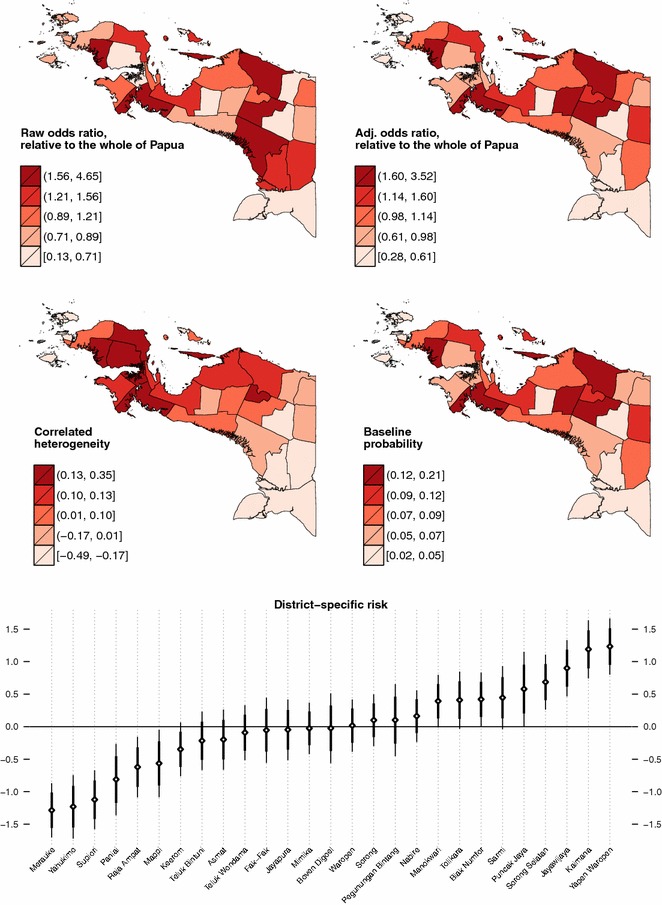
Estimated malaria risk in each district

**Fig. 8:**
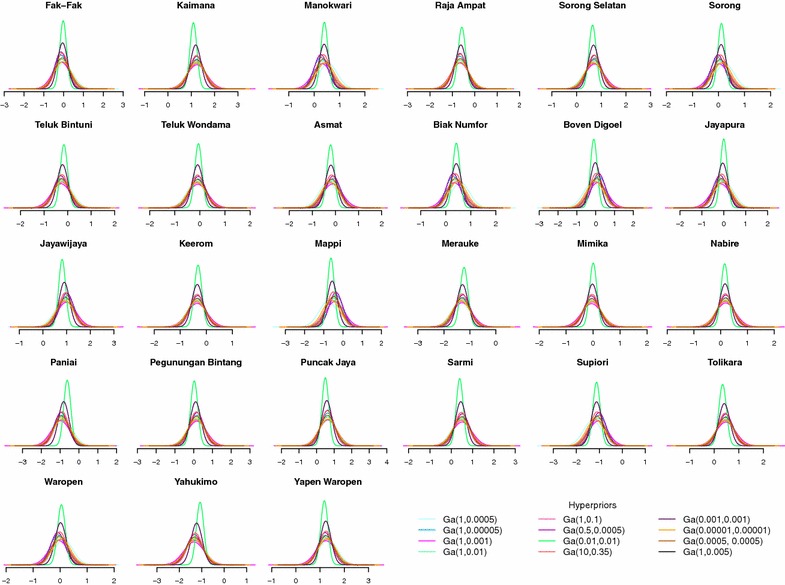
Posterior density of district-specific effects $(\xi _{j})$ under some alternative hyperprior specifications, logit scale

Having investigated the social and environmental correlates of malaria in Papua, we now turn our attention to Fig. 7, which shows the spatial distribution of the disease. The raw odds ratio $(\exp [\xi _{j}])$ displayed in the top-left panel shows where in Papua malaria is more prevalent (Null model), whereas the adjusted odds ratio shown in the top-right panel indicates which district has higher than expected prevalence *after* accounting for the effect of predictor variables in the Full model. It should be appreciated that, although the spatial patterning of malaria does not seem to vary that much between the two models, its variability is clearly reduced after the inclusion of the covariates. Apparent in the middle-left panel is the gradient of spatially correlated heterogeneity ($u_{j}$, in logit scale) that varies smoothly from the north-western side (high risk) to the south-eastern side (low risk) of the island. The middle-right panel re-expresses the estimated risk in terms of how likely, in the probability scale, the reference individuals are to be infected with malaria in each district $({\text {logit}}^{-1}[\beta _{0}+\xi _{j}])$. Finally, in the bottommost panel of the same figure, we rank the district-specific risk estimates ($\xi _{j}$, in logit scale) along with their 80 % (bold line) and 95 % (fine line) credible intervals. It is evident from these plots that, net of differentials in observable characteristics, four districts have unambiguously higher-than-expected malaria risks (Yapen Waropen, Kaimana, Jayawijaya, and Sorong Selatan). However, it is only when we apply Richardson’s criterion [75] to the posterior probability distributions $(\Pr [\exp \{\xi _{j}\}>1|{\mathbf {y}}])$ that we become aware of nine districts whose risks are deemed to be positively significant (Table 3). According to this criterion, clusters of elevated malaria risks are identified in north-central Papua, near Biak and Yapen islands, and around the north-western area. Figure 8 shows that this risk ranking exercise is robust to the assumption of hyperprior distributions.

**Table 3:** Risk category, based on Richardson et al. [75]

Positively significant$^{\mathrm{a}}$	Negatively significant$^{\mathrm{b}}$	Not significant$^{\mathrm{c}}$
Yapen Waropen	Merauke	Teluk Wondama
Kaimana	Yahukimo	Fak-fak
Jayawijaya	Supiori	Jayapura
Sorong Selatan	Paniai	Mimika
Biak Numfor	Raja Ampat	Boven Digoel
Puncak Jaya	Mappi	Waropen
Manokwari	Keerom	Pegunungan Bintang
Tolikara	Teluk Bintuni	Sorong
Sarmi	Asmat	Nabire

$$^{\mathrm{a}}$$ $${\text {Pr}}(\xi _j > 0 | {\mathbf {y}}) \ge 0.80$$

$$^{\mathrm{b}}$$ $${\text {Pr}}(\xi _j > 0 | {\mathbf {y}}) \le 0.20$$

$$^{\mathrm{c}}$$ $$0.20< {\text {Pr}}(\xi _j > 0 | {\mathbf {y}}) < 0.80$$

## Discussion

Analysing a subset of the largest public health data ever collected in Indonesia (National Basic Health Research 2007; N = 987,205), this study quantifies the district-specific risk of malaria in Papua and investigates how the disease is distributed across socio-demographic/economic strata. We predict the malaria status of 21,740 Papuans living in 27 districts using a Bayesian logistic regression model that accounts for the clustering of individuals within their areas of residence and the spatial autocorrelation among these locations. Both individual (age, sex, bed-net use, urban/rural) and contextual characteristics (elevation, forest density, median income) are included as predictors in the model.

In the analysis, a spatial gradient that varies smoothly from the north-western (higher risk) to the south-eastern (lower risk) areas of the island is identified; after taking this patterning into account, we then calculate, rank and map malaria risk in each district. We find that, even within this hyper-endemic island, the extent of spatial variation is *not* negligible. The model estimates that, while the baseline probability of malaria infection is about 2–5 % in the healthiest 20 % of districts, the figure can be as high as 12–21 % in the least healthy ones. This means that a typical male Papuan infant would have a 4–5 times higher probability of suffering from malaria if he were born in high-risk districts instead of in low-risk districts. Whether or not this inequality is acceptable within the current climate of Papua’s special autonomy [76] and Indonesia’s political decentralisation [20] is, of course, open to public debate.

Our risk mapping exercise further reveals three clusters of statistically significant high-risk districts located in north-central Papua (Sarmi, Tolikara, Puncak Jaya and Jayawijaya), near Biak and Yapen islands (Biak Numfor and Yapen Waropen), and around the north-western area of the island (Kaimana, Sorong Selatan and Manokwari). Because this risk ranking is independent of common socio-demographic/economic differentials and does seem to be robust to prior assumptions, health policy makers or planners may, therefore, want to conduct further epidemiological studies in these areas to unravel the possible social and environmental drivers of this excess risk. Furthermore, should there ever emerge an urgent need for allocating limited funds or human-capital resources in order to help local autonomous Papuan administrators achieve the country’s 2030 malaria elimination target [15], the Indonesian government could now consider utilising risk estimates and probabilistic maps presented in this study as a tool for prioritising development projects or other forms of intervention that may be funded by transfers from central to local governments. Such risk mapping activity is of high relevance for policy makers because the success of malaria control in many under-resourced countries often depends on targeted development of much-needed healthcare facilities in remote and sparsely populated areas [17, 77].

Independent of the aforementioned spatial effect, an elevated malaria risk is associated with living in rural areas, in densely forested districts, and in lowlands. This can be explained by the biology of the disease, as we have noted earlier. The literature suggests that these places provide not only a conducive environment for successful completion of the *plasmodium *growth-cycle [4, 30, 36, 37] but also a suitable breeding site and feeding ground for the *anopheles* vector [29, 38–40]. Small increases in malaria risk are also associated with being male and with being over age 55. These differentials may be driven by social norms with regards to gender roles and risk-exposure preferences [41, 45, 48, 78]—for instance, women (children) should stay safe at home while men (adults) have to work outside to provide for the family—although we ought to note that these effects may yet be confounded by the respondent’s immigration status. That non-native Papuans are more likely to seek malaria treatment and that they have lower acquired immunity to malaria due to their lack of exposure to infection are well-established in the literature [9, 12, 21, 77, 79]; unfortunately, information on individuals’ immigration status is unavailable in this particular survey data.

We further find that, even after adjusting for all these conventional risk factors, the risk of malaria in Papua remains far from evenly distributed by income level. Papuans living in the richest districts are estimated to have 38 % lower odds of having the disease than their peers in the poorest districts. So, if our reference infant were born in one of the richest districts, his estimated probability of being malaria-positive would be just 4 % instead of 6 %. This demonstrates that an income gradient in malaria prevalence indeed exists, even in Indonesia’s most deprived island group (recall Fig. 4). This finding is therefore consistent with the hypothesis that poverty creates conditions (poor housing, lack of knowledge, negative health behaviours) that favour the spread of infectious diseases and restrict access to prevention and treatment [5, 41–43].

Contrary to conventional wisdom, our analysis reveals that respondents’ use of ITN has a positive association with being malaria-positive. Initially, we suspected that this might be attributable to confounding or to an unaccounted data dependency structure in our naïve bivariate analysis. However, after fitting the fully specified multivariate model, the association persists. One plausible explanation is that a systematic bias due to differential item functioning (DIF) [80, 81] is at play, meaning that ITN users may over-report their illnesses simply because they are more aware of malaria symptoms than their non-user peers [44, 49, 82]. An equally plausible explanation is that this counter-intuitive result is actually an artefact of the targeted distribution of ITN to the less healthy sub-population, such that individuals who use ITN are actually those who have already been infected [83]. Another explanation, as documented in one ethnographic study from Malawi [42], is that economically disadvantaged individuals may use the net to enhance their outdoor income-generating activities (such as fishing), which could in turn, lead to increased risk exposure. Understanding which of these scenarios fits the reality in Papua is, indeed, a good motivation for future investigations.

The present study is not without limitations. One is that, due to a lack of data, we are unable to investigate how the prevalence of malaria varies by individual income and immigration status. Secondly, we are unable to estimate malaria risks in *Kota Sorong* and *Kota Jayapura* municipalities because their spatial polygons are not available. A more serious limitation, however, pertains to our use of clinical malaria data, which are fraught with measurement error. In the presence of DIF, clinical data could overestimate the true prevalence of malaria; but in hyper-endemic areas, they may just as easily underestimate the true prevalence because of the presumably high incidence of asymptomatic malaria [29, 49]. Somi et al. [82] point out that such measurement error, among other things, is often responsible for the attenuated estimates of socio-economic gradient in malaria prevalence (attenuation bias).

Despite these limitations, the present study still contributes to the literature in several ways. First, to the best of our knowledge, this study is among the first to provide a probabilistic characterisation of how malaria is distributed spatially *and* socially within Indonesian Papua. The Bayesian hierarchical modelling framework we adopt in this paper has proven to be useful and feasible for the purpose; policy makers could, therefore, consider employing it more routinely in the planning and evaluation of malaria elimination efforts in the country. The study is further distinguished in its use of randomly sampled population data, which have helped us contain to a large extent the threat of confounding and selection biases that limit the generalisability of existing community or facility studies [84]. Finally, the present study shows that in addition to being geography-dependent, malaria in Indonesian Papua is also a disease of poverty. A comprehensive malaria elimination programme in this region should therefore consider not only proximal factors impacting the biology of the *plasmodium* parasite and the *anopheles* vector but also distal socio-economic conditions facilitating malaria transmission [5, 29, 85, 86]. This means that classical health interventions via bed-net distribution, insecticide residual spraying, curative medication, and environmental controls should ideally be implemented alongside development programmes in the forms of job-creation, investment in education, income redistribution, and provision of affordable and accessible healthcare facilities [42, 87]. Unless the socio-economic factors that modulate the risk of infection are addressed, malaria elimination efforts in Papua will not be as effective as they are intended to be.

## Conclusion

This study is among the few to consider both the social and spatial distributions of malaria in Indonesian Papua, a hyper-endemic area that has received only little international attention due to the lack of data. The analysis shows that although malaria is geography-dependent in Papua, it is also a disease of poverty. This means that its eradication requires not only biological (proximal) interventions but also social (distal) ones. This finding is generally consistent with the body of literature linking socio-economic deprivation with malaria; it could, therefore, inform policy-making beyond Papuan context. This study also demonstrates the utility and feasibility of Bayesian hierarchical model for understanding the distribution of the infectious disease. Bayesian inference is still computationally more expensive than the classical one. However, as computing power continues to increase and new, efficient algorithms—such as one used in the present study—are devised, this flexible disease mapping technique could be employed more routinely in the planning and evaluation of malaria elimination efforts in other resource-poor settings.

## Abbreviations

WHO: World Health Organization
API: annual parasite incidence
PP: parasite prevalence
MAP: Malaria Atlas Project
mm: millimetre
Riskesdas: Riset Kesehatan Dasar
ITN: insecticide-treated net
Susenas: Survei Sosial Ekonomi Nasional
Podes: Potensi Desa
GADM: Global Administrative Area database
GLM: generalised linear model
CAR: conditional autoregressive
MRF: Markov random field
INLA: integrated nested Laplace approximation
MCMC: Markov Chain Monte Carlo
DIC: deviance information criterion
USD: US Dollar
IDR: Indonesian Rupiah
SD: standard deviation
CI: credible interval
OR: odds ratio
DIF: differential item functioning
